# Prognosis of persistent mitral regurgitation in patients undergoing transcatheter aortic valve replacement

**DOI:** 10.1007/s00392-020-01618-9

**Published:** 2020-02-18

**Authors:** Victor Mauri, Maria I. Körber, Elmar Kuhn, Tobias Schmidt, Christian Frerker, Thorsten Wahlers, Tanja K. Rudolph, Stephan Baldus, Matti Adam, Henrik ten Freyhaus

**Affiliations:** 1 Department of Cardiology, Heart Center, University of Cologne, Kerpener Str. 61, 50937 Cologne, Germany; 2 Department of Cardiothoracic Surgery, Heart Center, University of Cologne, Cologne, Germany; 3 Department of General and Interventional Cardiology, Ruhr-University Bochum, Heart- and Diabetes Center Nordrhein-Westfalen, Bad Oeynhausen, Germany

**Keywords:** TAVR, Mitral regurgitation, Aortic stenosis

## Abstract

**Objective:**

The objective of this study was to assess imaging predictors of mitral regurgitation (MR) improvement and to evaluate the impact of MR regression on long-term outcome in patients undergoing transcatheter aortic valve replacement (TAVR).

**Background:**

Concomitant MR is a frequent finding in patients with severe aortic stenosis but usually left untreated at the time of TAVR.

**Methods:**

Mitral regurgitation was graded by transthoracic echocardiography before and after TAVR in 677 consecutive patients with severe aortic stenosis. 2-year mortality was related to the degree of baseline and discharge MR. Morphological echo analysis was performed to determine predictors of MR improvement.

**Results:**

15.2% of patients presented with baseline MR ≥ 3 +, which was associated with a significantly decreased 2-year survival (57.7% vs. 74.4%, *P* < 0.001). MR improved in 50% of patients following TAVR, with 44% regressing to MR ≤ 2 +. MR improvement to ≤ 2 + was associated with significantly better survival compared to patients with persistent MR ≥ 3 +. Baseline parameters including non-severe baseline MR, the extent of mitral annular calcification and large annular dimension (≥ 32 mm) predicted the likelihood of an improvement to MR ≤ 2 +. A score based on these parameters selected groups with differing probability of MR ≤ 2 + post TAVR ranging from 10.5 to 94.4% (AUC 0.816; *P* < 0.001), and was predictive for 2-year mortality.

**Conclusion:**

Unresolved severe MR is a critical determinant of long term mortality following TAVR. Persistence of severe MR following TAVR can be predicted using selected parameters derived from TTE-imaging. These data call for close follow up and additional mitral valve treatment in this subgroup.

**Graphic abstract:**

Factors associated with MR persistence or regression after TAVR 
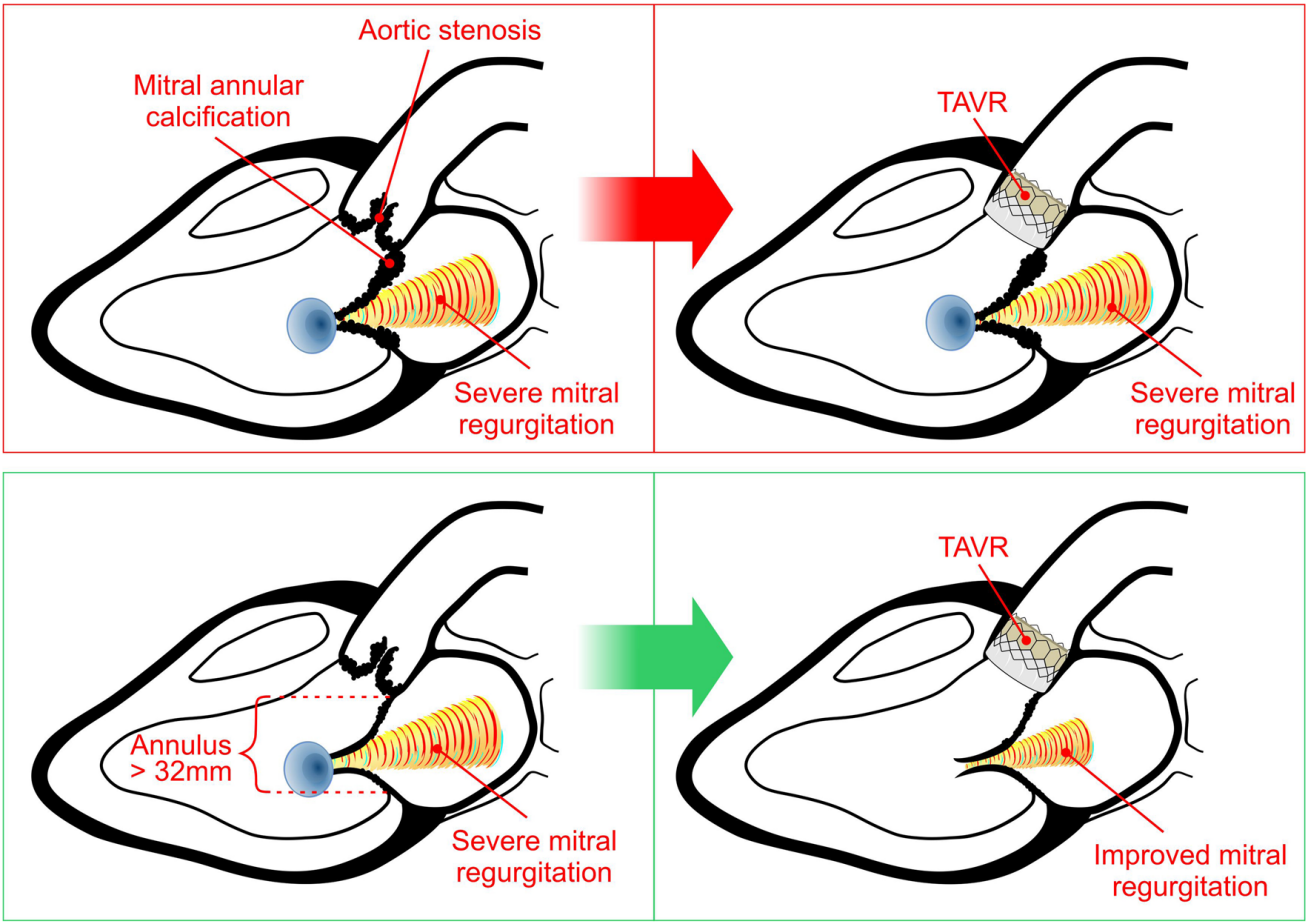

**Electronic supplementary material:**

The online version of this article (10.1007/s00392-020-01618-9) contains supplementary material, which is available to authorized users.

## Introduction

Relevant concomitant mitral regurgitation (MR) is present in up to one-third of patients with severe aortic stenosis [1–4]. However, data on multi-valve disease are scarce and, as a result, US- and European guideline recommendations for the management of multi-valve disease are limited [5, 6]. In surgical patients, there is a general consensus that in the presence of severe MR double-valve surgery is indicated, whereas the treatment of concomitant moderate MR is unclear. Combined aortic and mitral valve surgery yielded good long term functional results at the cost of a substantially increased operative mortality [7–9]. Over the last decade, transcatheter aortic valve replacement (TAVR) has evolved to clinical standard for the treatment of severe aortic stenosis in patients with increased risk for conventional surgery [5, 6]. In contrast to surgery, concomitant MR is typically left untreated at the time of TAVR, given that MR severity has been reported to decrease in surgical patients [4, 10]. However, MR remains unchanged or even worsens in some patients and predictors for MR improvement are not well defined [2, 3]. This is important since significant baseline or residual MR is associated with an increased mortality after TAVR [3, 4]. So far, however, it is unclear, (a) whether MR improvement after TAVR impacts on survival and (b) whether echocardiographic parameters can predict the resolution and persistence of MR in this patient population, respectively. Thus, the objective of this study was to assess the impact of MR improvement on long-term outcome and to determine underlying imaging predictors in patients with MR undergoing TAVR.

## Methods

836 consecutive patients underwent TAVR for severe native aortic stenosis between January 2013 and August 2016 at Cologne University Heart Center. 59 patients were excluded due to missing TTE data at the time of analysis (*n* = 48), previous mitral valve surgery (*n* = 3) or subsequent transcatheter mitral valve repair (*n* = 8). 777 patients with complete baseline TTE data were available for outcome analyses, and 677 with baseline and discharge TTE data were used for the analysis of MR evolution after TAVR (Supplementary Fig. 1). The study was approved by the institutional review board. All patients were considered not suitable for surgical aortic valve replacement by an interdisciplinary heart-team. Clinical and safety endpoints are reported according to the VARC-2 consensus.

### Assessment of MR severity and mitral valve complex morphology

The degree of MR was evaluated at baseline and post TAVR at discharge by two experienced echo readers unaware of clinical data and outcome measures using a multiparametric approach according to current recommendations integrating color doppler flow, vena contracta, effective regurgitation orifice area, pulmonary vein flow, mitral inflow pattern and velocity, indexed left atrial volume, left ventricular ejection fraction, and right ventricular systolic pressure [11–13]. Thereby, vena contracta was defined as the narrowest part of the MR jet and averaged in two planes (apical 2- and 4-chamber view). Left ventricular ejection fraction was calculated with biplane Simpson’s method. MR was graded integrating the aforementioned parameters as no/trace, mild (1 +), mild to moderate (2 +), moderate to severe (3 +) and severe (4 +). Disagreement about MR grades was resolved by consensus after evaluation by a third reader. MR improvement was defined as an improvement of at least one grade at discharge compared to pre TAVR. The morphological evaluation of the mitral valve apparatus included (a) identification of structural alterations, (b) extent and localization of leaflet calcification, and (c) extent of mitral valve annular calcification. As structural alterations were considered flail leaflet, prolapse (defined as systolic displacement of the mitral leaflet into the LA of at least 2 mm from the mitral annular plane in the parasternal long-axis view), perforation or cleft/indentation. Annular and leaflet calcifications were evaluated semi-quantitatively as shown in Supplementary Table 1. Annulus diameter was measured at mid-diastole in parasternal long-axis view. Based on these variables, a score to predict MR ≤ 2 + post TAVR was developed using an iterative approach with repetitive ROC curve analyses.

### Statistics

Categorical variables are reported as frequencies and percentages, continuous variables as means ± standard deviation. Differences between groups were evaluated using Fisher’s exact test for categorical variables and Student’s *t* test or Mann–Whitney *U* test for continuous variables, depending on their distribution. The Kruskal–Wallis test was used to test for differences of more than two groups. Kaplan–Meier curves were drawn for mortality at 2 year follow-up and compared using the log-rank test. Cox proportional hazards model was used to adjust for baseline characteristics. Repetitive ROC curve analysis was performed for evaluation of the prediction model. Two-sided *p* values < 0.05 were considered statistically significant. All analyses were performed using IBM SPSS Statistics version 22.

## Results

### Patient population

Patient characteristics are presented in Table 1. Mean age was 81.6 ± 6.3 years, 52% were female and patients had an intermediate to high risk for surgery (EuroSCORE II 4.8 ± 4.0%) and relevant comorbidities.

**Table 1:** Baseline patient characteristics

	No MR/MR ≤ 2 +	MR ≥ 3 +	*P* value
	*n* = 666	*n* = 111	
Age (years)	81.3 ± 6.4	83.3 ± 5.7	0.002
Female sex	342 (51.4)	65 (58.6)	0.096
BMI (kg/m^2^)	27.0 ± 5.1	25.0 ± 3.9	< 0.001
Coronary artery disease	421 (63.2)	66 (59.5)	0.459
Previous cardiac surgery	146 (21.9)	25 (22.5)	0.902
Peripheral artery disease	180 (27.0)	29 (26.1)	0.908
COPD	150 (22.5)	22 (19.8)	0.621
Diabetes mellitus	221 (33.2)	29 (26.1)	0.154
Arterial hypertension	617 (92.6)	103 (92.8)	1000
Atrial fibrillation	292 (43.9)	70 (61.3)	< 0.001
GFR (ml/min)	53 ± 24	39 ± 18	< 0.001
EuroSCORE II	4.5 ± 3.7	6.9 ± 5.2	< 0.001

Values are mean ± SD or *n* (%)

*BMI* body mass index, *COPD* chronic obstructive pulmonary disease, *GFR* glomerular filtration rate

### Evolution of MR

**Fig. 1:**
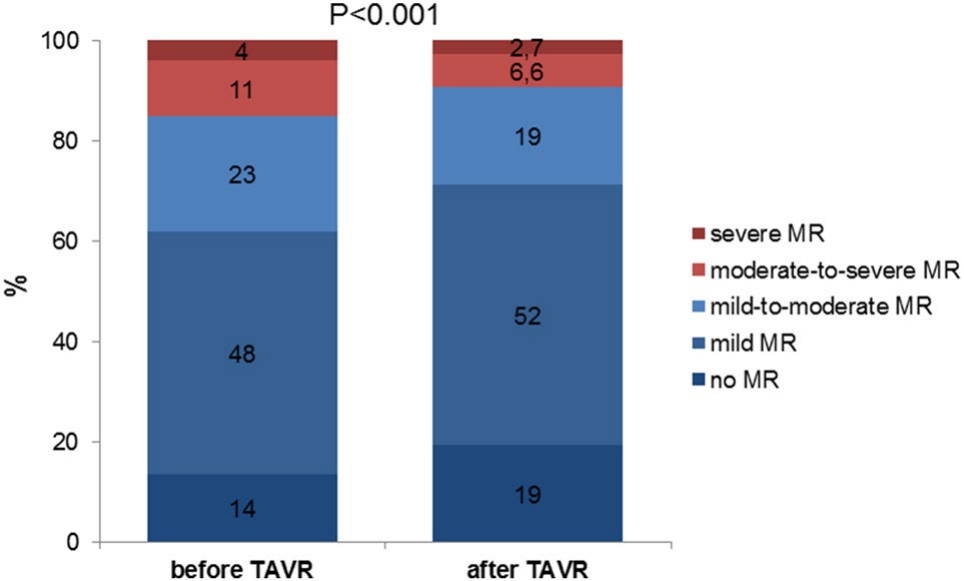
Change in MR-severity following TAVR. The prevalence of concomitant MR ≥ 3 decreased from 15.2 to 9.3% following TAVR (*P* < 0.001)

At baseline, 84.8% of patients presented with MR ≤ 2 + (13.6% no MR, 48.3% mild MR, 22.9% mild to moderate MR), and 15.2% with MR ≥ 3 + (11.2% moderate to severe MR, 4.0% severe MR). After TAVR, 90.7% of patients had MR ≤ 2 + (19.2% no MR, 52.0% mild MR, 19.5% mild to moderate MR), and 9.3% MR ≥ 3 + (6.6% moderate to severe MR, 2.7% severe MR; *P* < 0.001; Fig. 1). Echocardiographic parameters according to MR severity are shown in Table 2. Higher MR degrees were associated with lower LVEF, lower aortic mean gradient and higher systolic pulmonary artery pressure.

**Table 2:** Echocardiographic parameters according to degree of MR

	no MR		MR 1 +		MR 2 +		MR 3 +		MR 4 +		*P* value
	*n* = 113		*n* = 379		*n* = 174		*n* = 80		*n* = 31		
Vena contracta width (mm)	n/a		n/a		4.3 ± 0.9		5.9 ± 1.0		8.0 ± 1.3		< 0.001
E wave velocity (cm/s)	n/a		n/a		105 ± 24		135 ± 42		145 ± 54		< 0.001
LA volume index (ml/m^2^)	n/a		n/a		53.2 ± 15.8		62.0 ± 19.1		81.5 ± 50.3		0.001
PAPsys (mmHg)	37 ± 16		43 ± 17		46 ± 14		56 ± 15		59 ± 15		< 0.001
Mean aortic pressure gradient (mmHg)	45 ± 13		46 ± 16		45 ± 15		41 ± 15		33 ± 12		< 0.001
Aortic valve area (cm^2^)	0.75 ± 0.18		0.72 ± 0.19		0.67 ± 0.19		0.65 ± 0.23		0.77 ± 0.21		< 0.001
Ejection fraction (%)	54 ± 8		53 ± 9		48 ± 11		47 ± 11		45 ± 13		< 0.001

*LA* left atrium, *PAPsys* systolic pulmonary artery pressure

For further analyses, patients were dichotomized into groups with MR ≤ 2 + and MR ≥ 3 +. Patients with MR ≥ 3 + were significantly older, had lower left ventricular ejection fraction, lower glomerular filtration rate and presented more frequent with atrial fibrillation, resulting in a significantly higher EuroSCORE II (Table 1).

### Impact of concomitant MR at baseline on 2-year outcome

**Fig. 2:**
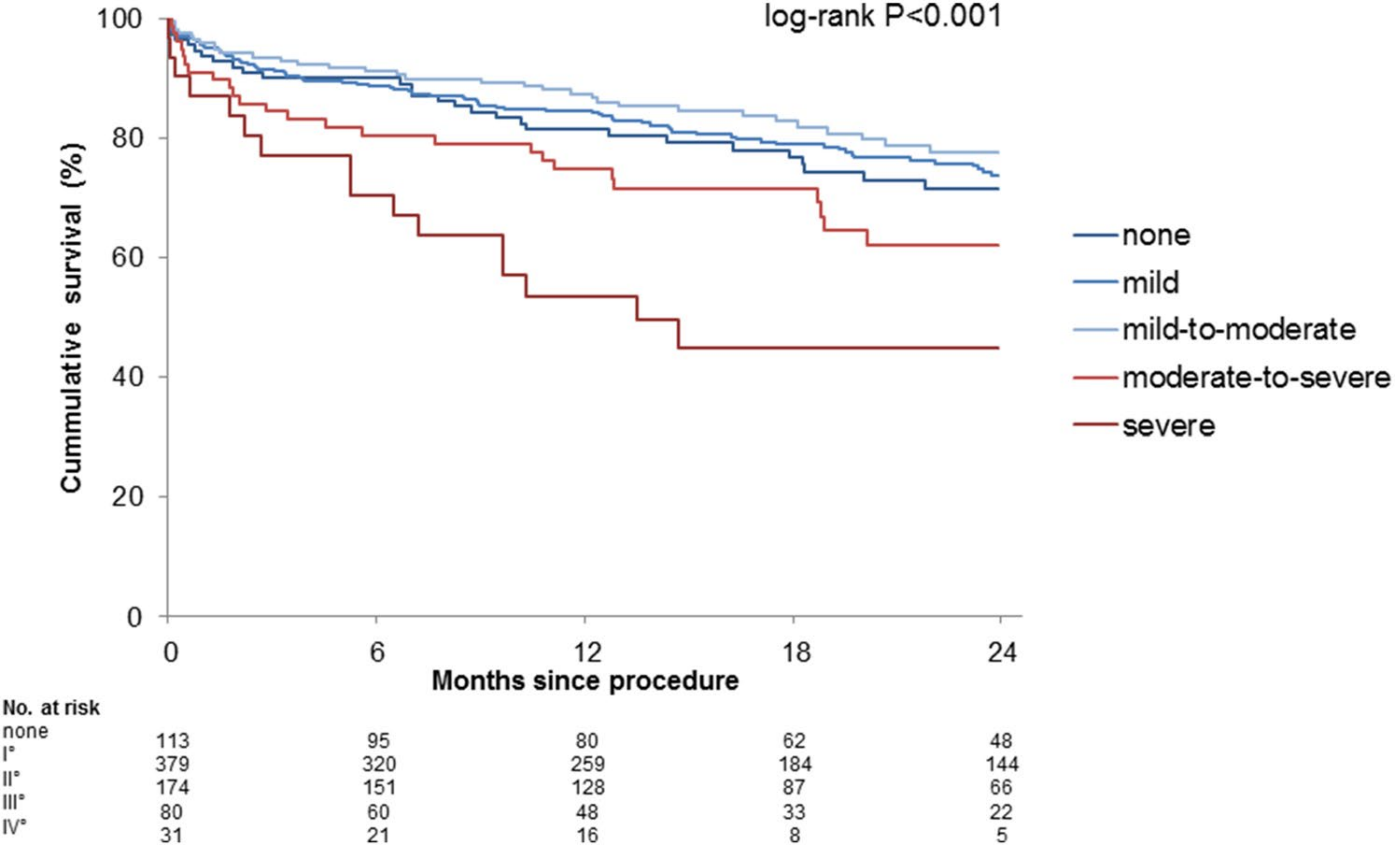
Prognostic relevance of baseline MR on mortality. The degree of baseline MR was significantly related to 2-year-mortality with higher mortality in patients with MR ≥ 3 + /4

The presence of concomitant moderate to severe or severe MR at baseline was significantly related to mortality at 2 years (Fig. 2). Estimated survival was 74.4% in patients with MR ≤ 2 +, and 57.7% in patients with MR ≥ 3 + at baseline, respectively [unadjusted HR 2.02 (95% CI 1.43–2.86); log-rank *P* < 0.001], and this difference persisted after adjustment for patient characteristics including sex, LVEF, chronic kidney disease, COPD, peripheral artery disease, atrial fibrillation, early safety, and EuroSCORE II [adjusted HR 1.70 (95% CI 1.17–2.48); *P* = 0.006].

### Evolution of MR in patients with MR ≥ 3 + 

**Fig. 3:**
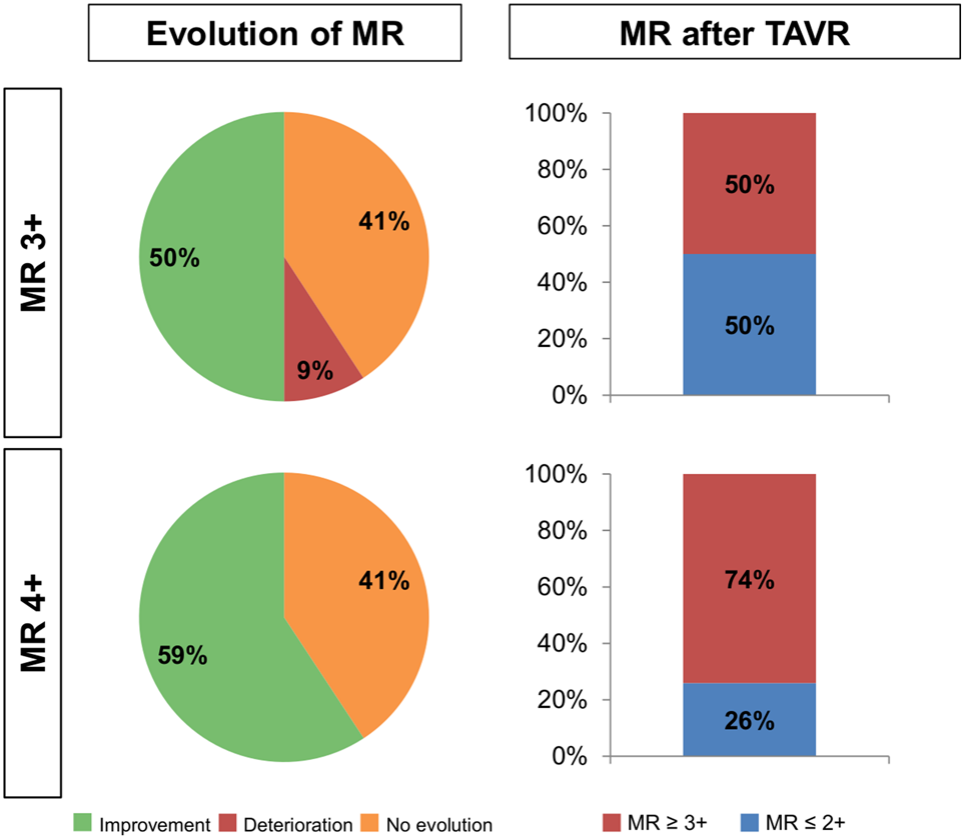
Evolution of MR in patients with baseline MR ≥ 3 + /4

103 patients presenting with MR ≥ 3 + had complete TTE data at baseline and discharge. Thereof, 76 patients had moderate to severe MR (73.8%), and 27 severe MR (28.2%). MR etiology was classified as degenerative, functional and mixed in 51.5%, 17.5% and 31.1% of patients, respectively. Of the 76 patients with moderate to severe MR, 38 (50.0%) experienced MR improvement ≥ 1° at discharge, whereas the degree of MR remained stable in 31 (40.8%) and worsened in 7 (9.2%) patients (average change − 0.59°). Consequently, MR at discharge was ≤ 2 + in 38 patients (50.0%) and remained ≥ 3 + in the other half of patients. Of the 27 patients with severe concomitant MR at baseline, 16 (59.3%) experienced MR improvement, and 11 (40.7%) did not (average change − 0.93°). As a result, MR was ≤ 2 + in 7 patients (25.9%) and remained ≥ 3 + in 20 (74.1%) after TAVR (Fig. 3). There were no statistically significant differences between patients with and without MR improvement after TAVR with respect to age, baseline comorbidities, EuroSCORE II, valve type (balloon-expandable vs. self-expanding), and conduction abnormalities (Supplementary Table 2), although atrial fibrillation was numerically more common among patients with persistent relevant MR.

**Fig. 4:**
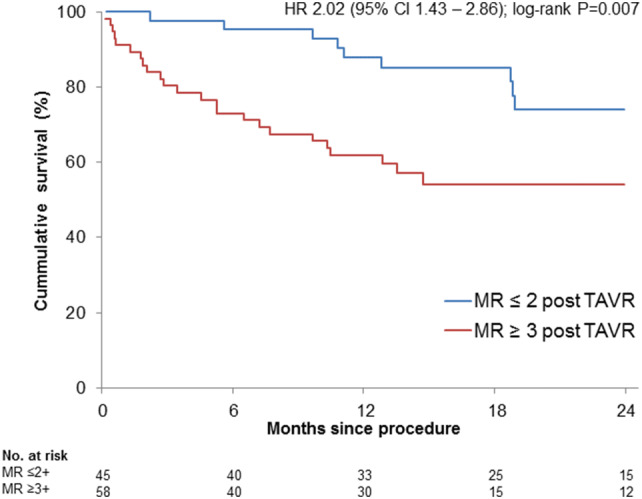
2-year-mortality in relation to MR-response following TAVR. The excess mortality in patients with baseline MR ≥ 3 + was driven only by patients with persistent MR ≥ 3 + post TAVR, whereas patients with improvement to MR ≤ 2 + had a mortality comparable to the overall-cohort

The regression of MR severity to MR ≤ 2 + after TAVR was associated with a significantly higher estimated 2-year survival compared with patients with remaining MR ≥ 3 + after TAVR [74.0% vs. 54.1%; HR 2.02 (95% CI 1.43–2.86); log-rank *P* = 0.007; Fig. 4], also after adjustment for patient characteristics including sex, LVEF, chronic kidney disease, COPD, peripheral artery disease, atrial fibrillation, early safety, and EuroSCORE II [adjusted HR 3.45 (95% CI 1.48–8.03); *P* = 0.004]. The survival of patients with regression to MR ≤ 2 + was comparable to the estimated survival of the overall cohort (77.8%). In the overall cohort, the estimated 2-year survival of patients with MR 3 + and MR 4 + was 59.0% and 51.9%, respectively. In the subgroup of 18 patients with functional MR, 9 presented with MR ≤ 2 + post-TAVR, and 9 patients had persistent MR ≥ 3 + post-TAVR. Patients with the improvement of their functional MR had a significantly better outcome compared to patients with MR persistence (2-year survival 80% vs. 28%; *P* = 0.021).

### Echo parameters predicting MR ≤ 2 + after TAVR

**Fig. 5:**
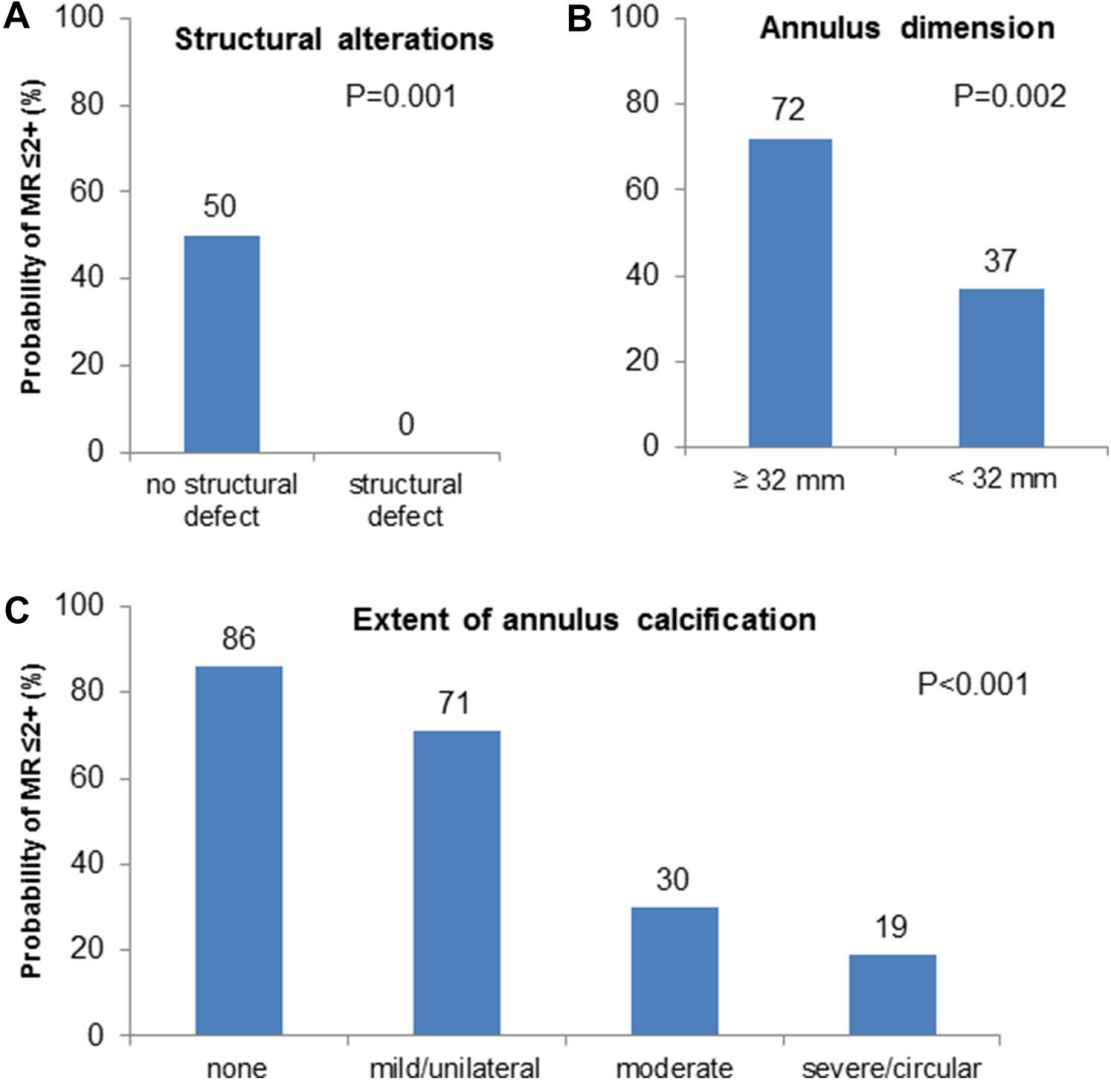
Morphological characteristics predicting MR regression to grade ≤ 2 +. **a** Structural alterations; **b** annulus dimension; **c** annulus calfication

A morphological characterization of the mitral valve apparatus was performed in patients with MR ≥ 3 + including identification of structural alterations, extent and localization of leaflet calcification, the extent of annulus calcification, and the dimension of the mitral annulus. Subsequently, the association of morphological characteristics with regression to MR ≤ 2 + was analyzed (Fig. 5a–c). As expected, the probability of regression to MR ≤ 2 + was 0% in patients with structural alterations (flail, prolapse, perforation) of the mitral valve compared to 50% in patients without (*P* < 0.001; Fig. 5a). Furthermore, the extent of annular calcifications was significantly inversely related to the probability of regression to MR ≤ 2 + post TAVR (no calcification: 86%; mild/unilateral: 71%; moderate: 30%; severe/circular: 19%; *P* < 0.001; Fig. 5c), whereas only a trend was observed for extent and location of leaflet calcifications (Supplementary Fig. 2). Also a larger mitral annulus was associated with a significantly higher probability of MR regression to ≤ 2 +, with a cut off for annular diameter of ≥ 32 mm as identified by ROC-curve analysis (72% vs. 37%, *P* = 0.002; Fig. 5b).

**Fig. 6:**
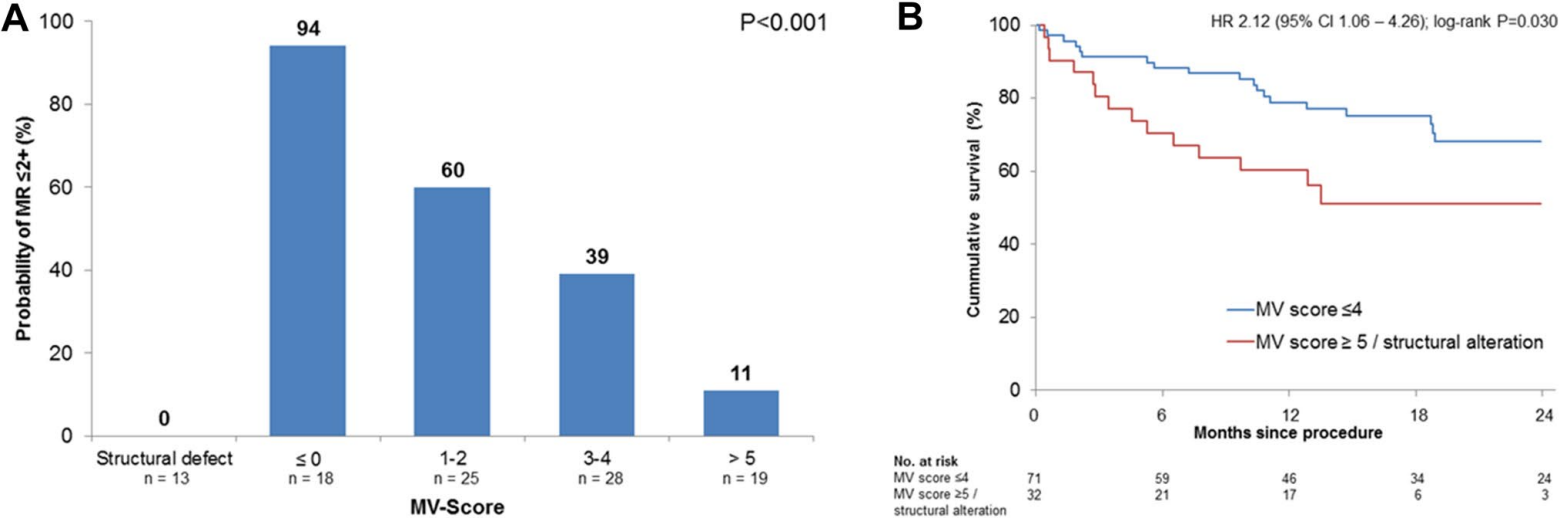
**a** MR reduction dependent on MV score. **b** Mortality dependent on MV score

Based on these analyses a mitral valve score was developed to predict the probability of MR improvement to MR ≤ 2 + after TAVR in patients without structural valve defects, integrating the degree of MR pre TAVR, the extent of annulus calcification and annular dimension. Due to the high correlation of annular and leaflet calcification, leaflet calcification was not included in the model. The score (Table 3) significantly predicts the probability of MR regression to MR ≤ 2 + [AUC 0.816 (95% CI 0.731–0.902), *P* < 0.001; Supplementary Fig. 3]. The probability of MR ≤ 2 + was 94.4% in patients with a score ≤ 0 and declined gradually to 10.5% in patients with a score ≥ 5 (*P* < 0.001, Fig. 6a). Structural valve alterations or a score ≥ 5 were associated with a significantly higher 2-year-mortality compared to patients with a MV score ≤ 4 [47.8% vs. 31.9%; HR 2.12 (95% CI 1.06–4.26); log-rank *P* = 0.030; Fig. 6b].

**Table 3:** MR reduction score to predict MR ≤ 2 +

Item	Points
MR 4 + at baseline	3
Extent of annulus calcification	
Mild/unilateral	1
Moderate	3
Severe/circular	5
Dimension of MV annulus	
< 32 mm	0
≥ 32 mm	− 2

## Discussion

The present study sought to evaluate the impact of MR improvement on 2-year survival and associated echo parameters in patients undergoing TAVR. The main findings of our study are (a) concomitant baseline MR ≥ 3 + is an independent predictor of 2-year mortality; (b) the degree of MR improved in 50% of patients, with MR graded ≤ 2 + in 44% after TAVR; (c) the significantly higher mortality of patients with MR ≥ 3 + was driven by patients with remaining MR ≥ 3 + after TAVR, whereas patients with MR reduction to ≤ 2 + had a 2-year-mortality comparable to the overall cohort; (d) TTE-based parameters with the ability to predict MR improvement and mortality following TAVR have been identified.

The prevalence of concomitant moderate/severe MR in patients with aortic stenosis has been shown to reach 30% [1–4, 14]. There are heterogenous reports characterizing the impact of concomitant MR on late mortality after TAVR. Although several studies suggested an association of relevant baseline MR and higher 30-day and 1-year-mortality, other studies did not [1, 3, 15–18]. In the present study, moderate-to-severe or severe MR at baseline was associated with significantly lower 2-year-survival, whereas mild-to-moderate MR was not. In line with many previous reports, MR improvement of at least 1 degree was observed in 50% of patients with MR ≥ 3 + at baseline [4, 14]. However, despite MR improvement in half of the patients, 56% of patients had persistent MR ≥ 3 +. Whether MR improvement after TAVR is associated with better survival is still a matter of debate, since most previous studies focused on the impact of *baseline* MR and did not evaluate the impact of MR improvement. Here, we show an association of MR persistence and mortality after TAVR: The significantly increased 2-year-mortality of patients with baseline MR ≥ 3 + was driven only by patients who remained at MR ≥ 3 + after TAVR, whereas patients with MR reduction to ≤ 2 + had a 2-year-mortality comparable to the overall cohort. In contrast, patients with severe MR at baseline in whom MR did not improve beyond MR 3 + remained at an increased risk for death despite MR improvement. This may explain the contrary results of a previous study which reported a lack of association of MR regression and improved survival, using a 3-class scale for MR assessment without differentiation between mild-to-moderate and moderate-to-severe MR [1]. In another study, Cortés and colleagues also did not find a link between MR improvement of 1 degree and 6-months mortality, however, reported—similar to our results—a trend towards increased cardiac mortality in patients with remaining MR ≥ 3 + at only 6-months of follow-up (24.4% vs. 15.7%; *P* = 0.151) [17]. Thus, MR improvement itself seems not to be necessarily related to better outcomes, but rather the absolute degree of MR following TAVR [19]. Two recent studies reported improved survival in patients with MR regression to none/mild MR following TAVR [18, 20]. However, both studies did not discriminate between mild to moderate and moderate to severe MR, a crucial graduation to dichotomize between favorable and poor prognosis when considering the current findings. Consequently, the relevant clinical question is not whether any improvement of MR after TAVR is achievable, but whether an improvement of MR severity to a degree ≤ 2 + is likely.

Several predictors of MR improvement including the absence of atrial fibrillation, prosthesis type and MR etiology have been described, but their causative impact remains controversial [10, 21]. Especially the simplified dichotomization of MR etiology into primary and functional origin does not appreciate the large individual variability of MR morphology and etiology. For instance, patients with primary MR present with a broad spectrum of different pathologies with likely different response to AVR. Similarly, functional MR due to malcoaptation in a dilated ventricle will likely show a different response to TAVR than MR due to a restrictive posterior leaflet cause by a regional wall motion abnormality. Moreover, in this specific patient population with a high burden of ischemic heart disease, degenerative structural alterations of the mitral valve apparatus and various ventricular remodeling processes a high prevalence of mixed MR etiologies is likely and reflected by the high number of mixed etiology in our and other cohorts [22]. This uncertainty may account for the highly variable prevalence of functional and degenerative MR in TAVR studies and contrary results regarding the value of MR etiology in predicting MR improvement and survival [2, 4, 17]. Consequently, objective imaging parameters are needed for a detailed morphological description of the mitral valve apparatus. Several smaller studies proposed different imaging features suggestive of functional MR including greater tenting area and larger left ventricular dimensions as predictors or MR improvement [21–24]. Similarly, in our study a larger mitral annulus was a predictor of MR improvement. The relatively low threshold of 32 mm as identified by ROC-curve analysis may reflect subtle enlargement of the mitral annulus as a response to elevated left ventricular pressures seen in aortic stenosis. Mitral annular calcification (MAC), suggestive of advanced degenerative valve disease, is associated with restricted leaflet motion, reduced annular contraction and subsequent valvular dysfunction [25, 26]. MAC was previously noted as a predictor of MR persistence after TAVR [17, 22, 27] and as an independent predictor of mortality [28]. Similarly, in our study, MAC as assessed by TTE was a relevant predictor of MR persistence. Extensive calcifications may alter the ability of reverse remodeling, and thus contribute substantially to MR persistence. Finally, as expected, severe structural alterations of the mitral valve were associated with MR persistence in all cases. Based on the imaging parameters mentioned above, a simple TTE-based evaluation score was developed which predicted the regression to MR ≤ 2 + with adequate accuracy represented by an AUC of 0.816. This score incorporates imaging features associated with both primary and secondary MR and thus acknowledges the high prevalence of mixed etiologies in this specific patient population.

In the context of expanding TAVR indications [29, 30], patients with concomitant valve disease warrant better prediction of the course of mitral regurgitation, procedural planning and post-procedural surveillance. Therefore, a careful evaluation of the mitral valve pathology in these patients is crucial. In particular, patients with remaining MR ≥ 3 + after TAVR may potentially benefit from additional transcatheter mitral valve treatment. Several reports demonstrated the safety and feasibility of a staged percutaneous approach [31, 32]. However, it should be noted that it remains unclear to date whether additional mitral valve treatment may actually result in improved survival [33]. Until data from randomized trials become available, a close follow up of patients and – if possible—concomitant treatment seems crucial in patients with remaining relevant MR.

### Study limitations

This study is of retrospective design, however, the overall size is the largest so far addressing the interplay between TAVR and the course of concomitant MR. Our analysis is based on discharge echocardiography, which may on the one hand underestimate the positive effect of TAVR in subsequent months. The fact however, that most patients were indicative of degenerative MR makes a marked additional improvement in MR severity unlikely.

## Conclusion

Concomitant MR is a frequent entity in the TAVR population associated with poor clinical outcome. We show that half of the patients with MR ≥ 3 + at baseline experience MR improvement after TAVR, however, only MR regression to MR ≤ 2 + is associated with significantly better survival. A simple TTE-based score may help to evaluate the likelihood of improvement and the necessity of additional mitral valve treatment, which may have important implications on prognosis in this patient cohort.

## Electronic supplementary material

Below is the link to the electronic supplementary material.

Supplementary file1 (DOCX 14 kb)

Study flow chart (TIF 20 kb)

Relation of leaflet calcification and MR regression. Extent and location of leaflet regression was not integrated into the final model due to a high correlation with annular calcification (TIF 94 kb)

ROC-curve for MV-Score. AUC 0.813 (95% CI 0.724-0.902); P<0.001 (TIF 71 kb)
