# Supplementary material for: Prognosis of persistent mitral regurgitation in patients undergoing transcatheter aortic valve replacement
Source: Clin Res Cardiol. 2020 Feb 18;109(10):1261–70. doi: 10.1007/s00392-020-01618-9 (PMC7515951; doi:10.1007/s00392-020-01618-9)
Supplement: Supplementary file 1 — Supplementary file1 (DOCX 14 kb) [file 392_2020_1618_MOESM1_ESM.docx]

## Supplementary Table 1: Semi-quantitative grading of annular and leaflet calcification

| Extent of leaflet calcification |
| --- |
| - none |
| - isolated spots |
| - larger spots interacting with leaflet motion |
| - severe with restrictive leaflet motion |
|  |
| Location of leaflet calcification |
| - none |
| - base or tips of leaflets |
| - base and tips of leaflets |
|  |
| Extent of annulus calcification |
| - none |
| - mild/unilateral |
| - moderate |
| - severe/circular |

## Supplementary Table 2: Characteristics of patients with baseline MR ≥3+ depending on MR regression

|  | **MR ≤2+** | **MR ≥3+** | **P** |
| --- | --- | --- | --- |
|  | n=45 | n=58 |  |
| Age | 82.9±6.0 | 83.7±5.5 | 0.442 |
| LVEF | 46±11 | 49±12 | 0.085 |
| GFR | 42±23 | 37±12 | 0.440 |
| Atrial fibrillation | 53.3% | 72.4 % | 0.062 |
| COPD | 15.6% | 22.4% | 0.457 |
| PAD | 22.2 % | 25.9 % | 0.817 |
| EuroSCORE II | 6.5±5.4 | 6.9±4.9 | 0.592 |
| PVR ≥moderate | 2.4% | 10.9% | 0.135 |
| LBBB (old and new) | 20.0 % | 18.9 % | 0.896 |
| PPM | 17.8 % | 17.2 % | 1.000 |
| Valve type |  |  |  |
| - BE | 55.6 % | 62.1 % | 0.548 |
| - SE | 44.4 % | 37.9 % |  |
| Early safety | 2.2 % | 15.5 % | 0.040 |
|  |  |  |  |
| **MV Score parameters** |  |  |  |
| MR 4+ at baseline | 15.6 % | 34.5 % | 0.042 |
| Extent of annulus calcification* |  |  | <0.001 |
| - mild/unilateral | 60.0 % | 27.6 % |  |
| - moderate | 20.0 % | 41.1 % |  |
| - severe/circular | 6.7 % | 25.9 % |  |
| Dimension of MV annulus |  |  | 0.001 |
| - < 32 mm | 51.1 % | 81.0 % |  |
| - ≥ 32 mm | 48.9 % | 19.0 % |  |

BE – balloon expandable; COPD – chronic obstructive pulmonary disease; GFR – glomerular filtration rate; LBBB – left bundle branch block; LVEF – left ventricular ejection fraction; MV – mitral valve; MR – mitral regurgitation; PAD – peripheral artery disease; PPM – permanent pacemaker; PVR – paravalvular regurgitation; SE – self-expanding
