# Supplementary figures and images for: Prognosis of persistent mitral regurgitation in patients undergoing transcatheter aortic valve replacement
Source: Clin Res Cardiol. 2020 Feb 18;109(10):1261–70. doi: 10.1007/s00392-020-01618-9 (PMC7515951; doi:10.1007/s00392-020-01618-9)

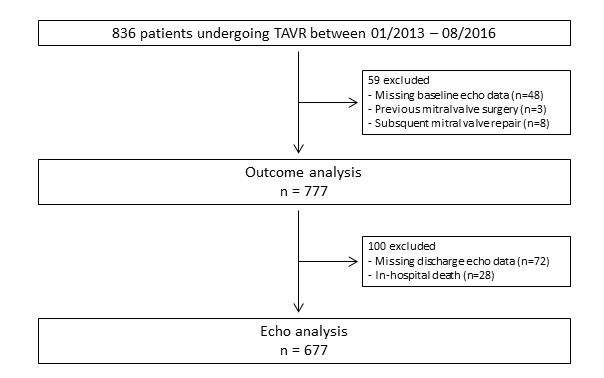

Supplement: Supplementary file 2 — Study flow chart (TIF 20 kb) [file 392_2020_1618_MOESM2_ESM.tif]

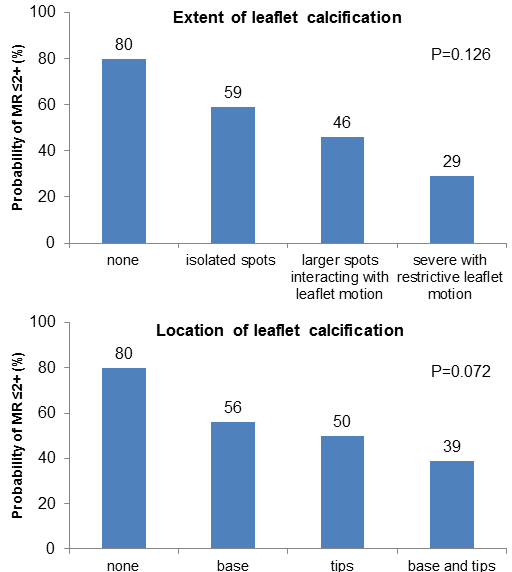

Supplement: Supplementary file 3 — Relation of leaflet calcification and MR regression. Extent and location of leaflet regression was not integrated into the final model due to a high correlation with annular calcification (TIF 94 kb) [file 392_2020_1618_MOESM3_ESM.tif]

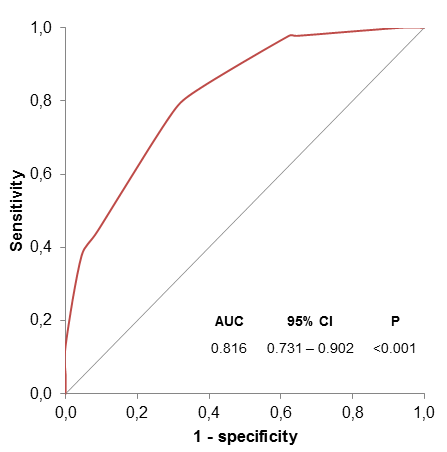

Supplement: Supplementary file 4 — ROC-curve for MV-Score. AUC 0.813 (95% CI 0.724-0.902); P<0.001 (TIF 71 kb) [file 392_2020_1618_MOESM4_ESM.tif]
